# Autoimmune Pancreatitis: A Review

**DOI:** 10.3390/jcm14093076

**Published:** 2025-04-29

**Authors:** Varun Vemulapalli, Cristina Natha, Anusha Shirwaikar Thomas

**Affiliations:** 1 Department of Internal Medicine, UT Houston Health Science Center, Houston, TX 77030, USA; varun.vemulapalli@uth.tmc.edu (V.V.); cristina.m.natha@uth.tmc.edu (C.N.); 2 Department of Gastroenterology, Hepatology, and Nutrition, The University of Texas MD Anderson Cancer Center, Houston, TX 77030, USA

**Keywords:** autoimmune pancreatitis, AIP, immune checkpoint inhibitor, IgG4-RD

## Abstract

Autoimmune pancreatitis is a rare condition of pancreatic inflammation with two classic subtypes. The emergence of a third subtype, ICI-induced pancreatitis, highlights the need for knowledge of each type to ensure accurate diagnosis and treatment. Abbreviations: AIP—Autoimmune pancreatitis; AIP-1—Type 1 autoimmune pancreatitis, also known as lymphoplasmacytic sclerosing pancreatitis (LPSP); AIP-2—Type 2 autoimmune pancreatitis, also referred to as idiopathic duct-centric pancreatitis (IDCP); AIP-3—Type 3 autoimmune pancreatitis, also known as immune checkpoint inhibitor (ICI)-induced autoimmune pancreatitis; IgG4-RD—Immunoglobulin G4-related disease.

## 1. Introduction

Autoimmune pancreatitis (AIP) is a rare, chronic form of pancreatitis that is associated with inflammation of the pancreas due to damage from an abnormal immune system response [1]. It is often clinically characterized by a frequent presentation of obstructive jaundice [2]. Imaging often may reveal enlargement of the pancreas and pancreatic duct/bile duct narrowing, mimicking pancreatic carcinoma [3], and histology is typically significant for a predominant lymphoplasmacytic infiltrate and fibrosis, which can lead to both endocrine and exocrine pancreatic dysfunction [4]. AIP has classically been divided into two main subtypes: Type 1 (AIP-1), also known as lymphoplasmacytic sclerosing pancreatitis (LPSP), and Type 2 (AIP-2), also called idiopathic duct-centric pancreatitis (IDCP). 

Type 1 autoimmune pancreatitis is a manifestation of IgG4-related disease (IgG4-RD) that primarily affects older males. It is marked by painless obstructive jaundice with the potential for systemic involvement of multiple sites, including salivary glands, thyroid, kidneys, and bile ducts [5,6]. Elevated serum IgG4 levels and histopathologic features, such as storiform fibrosis, obliterative phlebitis, and lymphoplasmacytic infiltration with IgG-4 plasma cells, are hallmarks of AIP-1 [7].

In contrast to AIP-1, AIP-2 is more commonly seen in younger adults and is not a systemic disease. It primarily affects the pancreas and has an association with inflammatory bowel disease (IBD), and typically ulcerative colitis [8]. Histologically, a fibroinflammatory process involving mainly pancreatic ducts, with a neutrophilic infiltration of medium and small ducts, is a key finding of AIP-2. However, the most distinctive feature is the presence of granulocytic epithelial lesions (GELs) in medium-size and small ducts [9].

Unlike classical AIP, type 3 AIP is a distinct drug-induced immune mediated chronic inflammatory disease of the pancreas secondary to immune checkpoint inhibitors (ICI) [10]. ICIs are monoclonal antibodies that function by upregulating effector T cell activation by inhibiting the pathways that moderate their production [11]. With increased T-cell production, the targeted destruction of tumor cells can occur, allowing for broad use of these drugs in cancer therapy. Differing from traditional chemotherapy and targeted therapy, ICI can break the state of immune tolerance in the tumor microenvironment and activate the body’s anti-tumor immunity. However, this increased immune response can become excessive and potentially result in inflammatory toxicities affecting different organ systems, known as immune-related adverse events (irAEs) [12].

The majority of patients with AIP-3 typically present with an asymptomatic elevation of pancreatic enzymes a few months after the initiation of ICI therapy [13]. While the exact mechanism of AIP-3 remains unclear, it is speculated that checkpoint inhibition induces a nonspecific inflammatory response mediated by T cells in the pancreas, resulting in pancreatic inflammation, acinar atrophy, and fibrosis in some cases [14]. Unlike AIP-1 and AIP-2, type 3 AIP has not been shown to have a response to treatment with corticosteroids [15]. As ICIs become more widely used in oncology, early recognition and awareness of ICI-induced AIP are crucial for prompt diagnosis and treatment.

## 2. Epidemiology

Despite an increasing awareness, and recognition of AIP, it remains a rare condition. Less than 2% of patients being evaluated for chronic pancreatitis are diagnosed with AIP, with one study showing more than 80% of these patients to have been diagnosed with AIP-1 [16]. Given the rarity of this disease, there is still little data on its prevalence in the United States. However, a study in Germany found the prevalence of AIP to be ~9% in individuals with nonalcoholic pancreatitis and to be 0% in those with alcoholic pancreatitis. The incidence was reported to be less than 1 in 100,000 individuals [17]. Furthermore, a 2016 study in Japan found a prevalence rate of 10.1 per 100,000 individuals and an incidence of 3.1 per 100,000 individuals [18]. Interestingly, a 2011 study in Japan yielded results of significantly lower rates, with a prevalence rate of 4.6 per 100,000 individuals and incidence of 1.4 per 100,000 individuals [19]. Both studies did report similar male-to-female ratios (2.94 and 3.2, respectively) and mean average ages (68.1 vs. 66.3, respectively), indicating that this condition primarily affects older men. Notably, studies have also revealed that AIP-1 is more common in eastern countries while AIP-2 is more common in Western countries [20] (Table 1).

With the relative paucity of data on AIP-3, it is difficult to know the incidence of this condition. However, some studies have estimated it to be up to 4% in those who have received ICI therapy [21,22,23]. In addition, AIP-3 is commonly associated with other irAEs, all of which are affected by the type of ICI administered and by type of malignancy. In fact, CTLA-4 inhibitors have been found to have a higher incidence (83%) of irAE than other ICIs. However, regarding the development of AIP-3 specifically, combination ICI therapy or monotherapy with PD-L1 were both found to have a higher incidence than monotherapy with CTLA-4 inhibitors alone [24]. Moreover, individuals with advanced stage malignancy are more likely to develop irAE. In one study, individuals with stage III or IV genitourinary cancer were the most likely to develop AIP-3 [25].

## 3. Pathogenesis

The pathogenesis of AIP is complex and likely to be multifactorial in nature. While genetic and environmental factors are theorized to contribute to this process, there is already adequate research to suggest that the immune system has a profound influence in the development of AIP.

In the early stages of AIP discovery and research, type 1 and type 2 AIP were named based on their histological characteristics. Early studies in AIP research found increased infiltration of IgG4 producing plasma cells and lymphocytes in pancreatic tissue, particularly in LPSP, although the mechanism behind this finding remains unclear [26]. This finding prompted further research that concluded that elevated serum levels of IgG4 are a hallmark of LPSP. Today, AIP-1 is also referred to as IgG4-related pancreatitis (IgG4-RP) and is included in a group of diseases referred to as IgG4-related diseases (IgG4-RD) [1]. IgG4-RD is a systemic, inflammatory disease, sometimes affecting multiple organs, with unclear etiology. Almost any organ can be involved, and the disease is also characterized by tissue infiltration of IgG4 positive plasma cells and elevated levels of serum IgG4. Interestingly, the histological findings of this disease are the same no matter which organ is affected [23]. Although IgG4 is thought to be the least inflammatory of the IgG subsets, its role in IgG4-antigen complex formation is thought to trigger an immune response that attracts macrophages, T cells, and other plasma cells, which will activate inflammatory pathways [27]. Furthermore, AIP-1 is characterized by activation of Th2-predominant T cells which secrete inflammatory cytokines, such as IL-4, IL-5, and IL-13, to further exacerbate inflammation in AIP-1 [28]. Additionally, IL-33, secreted by toll-like receptors (TLRs), is another pro-inflammatory cytokine that has been found to be elevated in AIP-1 due to TLR overexpression in the pancreas [29].

In addition to plasma cell infiltration of pancreatic tissue, the immune system plays a role in AIP-1 development through the upregulation of plasmacytoid dendritic cells (pDCs), a feature observed in various cases of IgG4-RD [29]. These cells are major contributors to circulating type 1 interferons (IFN-I), which play a significant role in microbial defense. However, inadequate regulation of IFN-I production contributes to the increased inflammation that characterizes AIP-1 [29]. In fact, animal studies have shown that an increase in pDCs is associated with the development of AIP, and inhibition of pDCs and IFN-I prevented this process [30]. Given these findings, IFN-I inhibition may have a role as therapeutic intervention for AIP-1 and IgG4-RD [30,31].

The pathogenesis of AIP-2, or idiopathic duct-destructive pancreatitis, is not as established as that of AIP-1. While little is known, research has shown that it is unrelated to IgG4 and different to AIP-1 in that regard. One process associated with AIP-2 is the upregulation of IL-8, a cytokine that induces the chemotaxis of neutrophils [29]. One study found elevated levels of IL-8 when compared to AIP-1 by both quantitative polymerase chain reaction and immunostaining of pancreatic tissue [32]. The recruitment of neutrophils to sites of inflammation by IL-8 likely contributes to the prominent infiltration of neutrophils in the epithelium of pancreatic ducts, a key histologic finding in AIP-2 [29]. Another proposed process is the activation of Th17, a subset of CD4 effector T cells, and the subsequent secretion of inflammatory cytokines. A study showed increased expression of Th17 cells when compared to chronic pancreatitis and normal pancreas levels [33]. While the findings of this study suggest the implication of Th17 cells in AIP-2 pathogenesis, the exact mechanism of cell infiltration of the pancreatic tissue is unclear.

Type 3 AIP is an uncommon irAE and, for this reason, has been understudied. Prior studies of other cases of irAE have established that ICIs work by targeting cytotoxic T-lymphocyte-associated antigen 4 (CTLA-4), programmed cell death protein 1 (PD-1), or programmed cell death ligand 1 (PD-L1). In doing so, they facilitate T cell proliferation, which can successfully treat malignancies but can also lead to an unmitigated T cell response that results in irAEs [11]. However, the exact mechanism of how activated T cells cause these injuries is not yet known. 

One potential explanation for the constellation of findings seen in AIP-3 is that the pancreatic injury could predominantly be a peri-acinar stromal injury with minimal collateral acinar injury [15]. This study suggested that progression to more acinar injury could prompt the emergence of a more typical presentation of pancreatitis with more evident radiographic findings. However, further studies would be needed to support this idea and elucidate the mechanism by which it would occur.

While AIP-3 is a relatively understudied irAE, there have been other studies on immune mediated colitis (IMC) and its similarities to inflammatory bowel disease (IBD). One such study revealed that CTLA-4 mutation or deletion in mice led to fatal multisystem inflammatory processes in some cases [34]. In humans, CTLA-4 polymorphisms were also found to increase the risk of developing IBD [35]. It has already been established in animal studies that CTLA-4 polymorphisms are associated with several human autoimmune disorders, with one study also observing significant pancreatic manifestations [36]. Similar to how CTLA-4 polymorphism predisposes an individual to IBD, it is possible that a similar relationship exists between CTLA-4 and pancreatitis in humans that is exacerbated in ICI therapy, resulting in AIP-3. Additionally, given that the incidence of AIP-3 is higher with the use of PD-L1 inhibitors than with other ICIs, it is reasonable to suggest that there could also be a similar relationship between PD-L1 polymorphism and pancreatitis. 

## 4. Clinical Presentation

AIP-1 predominantly affects older males, typically in the sixth decade of life. Painless obstructive jaundice is a common presenting feature of AIP-1 often resulting from concurrent bile duct involvement. Notably, AIP-1 is the pancreatic manifestation of IgG4-RD and rarely occurs in isolation. It is frequently accompanied by IgG4-related sclerosing cholangitis (IgG4-SC) and retroperitoneal fibrosis, reflecting the systemic nature of the disease [16,37,38]. Studies estimate that 60–90% of patients with AIP exhibit extra-pancreatic involvement, most commonly affecting the bile ducts, retroperitoneum, kidneys, lymph nodes, and salivary glands [7,26,39]. Recognizing this multisystem pattern is crucial, as extra-pancreatic signs such as jaundice, renal dysfunction, or lymphadenopathy may provide critical diagnostic clues and guide a more comprehensive evaluation. Interestingly, new-onset diabetes mellitus (DM) has also been associated with AIP-1. Previous reports have documented high prevalence of diabetes mellitus (DM) among patients with autoimmune pancreatitis (AIP), ranging from 21% to 83%, with 17% to 65% of patients developing DM concurrently with the onset of AIP [40]. Treatment with corticosteroids has been shown to improve insulin secretion and help with glycemic control in many of these patients [41,42]. However, it has also been observed to potentially aggravate the DM of some, particularly in older patients [43] (Table 1). 

In contrast to AIP-1, AIP-2 more commonly affects younger patients with a median age of 32.5 years and has no gender predilection. Unlike AIP-1, this subtype has a lower incidence of obstructive jaundice, is a more localized form of the disease, and often presents with abdominal pain and acute pancreatitis, affecting up to 50% of patients [44]. AIP-2 frequently coexists with IBD, particularly ulcerative colitis [45]. IBD diagnosis usually precedes AIP diagnosis by 2–5 years, and patients with pre-existing IBD often have active intestinal disease at the time of AIP diagnosis [46] (Table 1). 

AIP-3 occurs in patients receiving ICI therapy for a variety of malignancies. It often manifests three to four months after ICI exposure, even up to two years after a dose, and may present alongside other irAEs, such as colitis and thyroiditis [15,23]. The clinical presentation of AIP-3 can vary from asymptomatic pancreatic enzyme elevation, acute pancreatitis with abdominal pain, and/or incidental imaging evidence of pancreatitis [14]. Interestingly, it has been established that nearly two-thirds of patients with Type 3 AIP remain clinically asymptomatic [23]. Furthermore, like type 2 AIP, AIP-3 typically does not present with systemic involvement (Table 1).

## 5. Evaluation and Diagnosis

The Atlanta Criteria have long been considered the gold standard for both diagnosis and severity assessment of acute pancreatitis. Acute pancreatitis is diagnosed when a patient meets at least two of the following three criteria: acute epigastric pain, serum amylase or lipase levels elevated to at least three times the upper limit of normal, and radiologic evidence of pancreatic inflammation on CT, MRI, or ultrasound [47]. Due to the clinical manifestations of AIP often not fitting within the Atlanta criteria and the lack of universally accepted diagnostic guidelines, the diagnosis of AIP has been historically challenging.

Over the past decade, various diagnostic frameworks have emerged across Asia, Europe, and North America, reflecting differences in clinical practices and test availability. For example, Japan mandates endoscopic retrograde pancreatography, while Western clinicians avoid it due to pancreatitis risk. Similarly, pancreatic core biopsy is standard at the Mayo Clinic but is not widely adopted [2]. Given that AIP consists of two well established subtypes, separate diagnostic approaches are required. 

The International Consensus Diagnostic Criteria (ICDC) for AIP were formulated by combining the strengths of various criteria to facilitate accurate diagnosis and are currently the sole criteria that can identify AIP with differentiation between types 1 and 2 [48]. Diagnosis is based on five key features: pancreatic imaging, serology (IgG4 levels), other organ involvement, histopathology, and response to steroids. AIP is diagnosed as definitive or probable based on specific criteria combinations [49]. The ICDC standardizes AIP diagnosis, distinguishing it from pancreatic cancer and guiding appropriate treatment (Table 2).

While the ICDC for AIP-1 and AIP-2 rely on the above 5 features, AIP-3 is defined primarily by its association with ICI therapy and its clinical response to steroids. However, due to the lack of specific biomarkers or histological features distinguishing AIP-3 from other forms of AIP, clinical judgment and the context of ICI therapy are key to diagnosis (Table 2).

After diagnosis, the next step is to assess the severity of the disease. Several scales are used to do this, the most notable of which are the Common Terminology Criteria for Adverse Events (CTCAE) scale and the National Comprehensive Cancer Network (NCCN) scale. These scales assess the severity of pancreatitis and lipase elevation, while the Marshall scoring system is a different scale that accounts for the presence of organ failure [24]. While there is some overlap of the criteria between the scales, the widespread use of multiple scales highlights the need for a universal criterion to assess this disease.

### 5.1. Radiographic Findings

**Figure 1:**
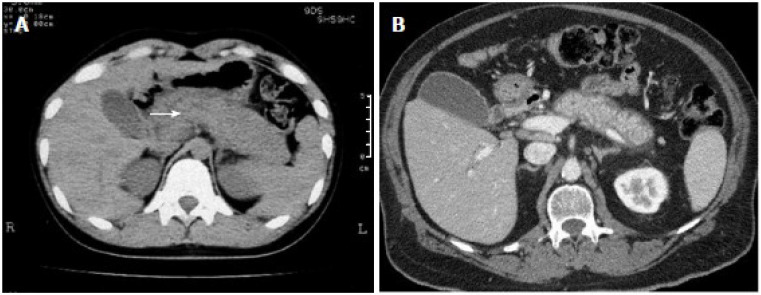
Characteristic Imaging Findings of AIP-1. (**A**) Diffuse pancreatic enlargement with the characteristic sausage-shaped appearance can be seen (arrow). (**B**) The peripancreatic rim seen around the body and tail of the pancreas is a key radiographic finding of AIP [52].

In AIP-1, pancreatic imaging typically reveals diffuse pancreatic enlargement, which is often associated with delayed enhancement following contrast administration. This enlargement is usually seen in the body or tail of the pancreas and may result in a “sausage-shaped” appearance and consequent loss of the typical lobular structure (Figure 1). Imaging may also reveal hallmark features of IgG4-related biliary involvement, including bile duct wall thickening and irregular narrowing of the pancreatic duct; however, this typically does not cause significant upstream duct dilation as seen in pancreatic cancer [3]. These findings are frequently associated with IgG4-SC, which may be radiographically indistinguishable from other forms of cholangiopathy, such as primary sclerosing cholangitis [26,39,50]. A peripancreatic rim, also referred to as a capsule-like rim, is another key imaging feature observed in AIP and can be useful in distinguishing it from cancer (Figure 1). Although it is most commonly seen in AIP-1, it may occasionally appear in AIP-2 [51] (Table 1).

The imaging findings are often similar in AIP-2, but this condition tends to be less associated with systemic manifestation, and pancreatic enlargement may not be as pronounced, or there may be focal or segmental pancreatic involvement, typically affecting the pancreatic tail, rather than diffuse enlargement. 

**Figure 2:**
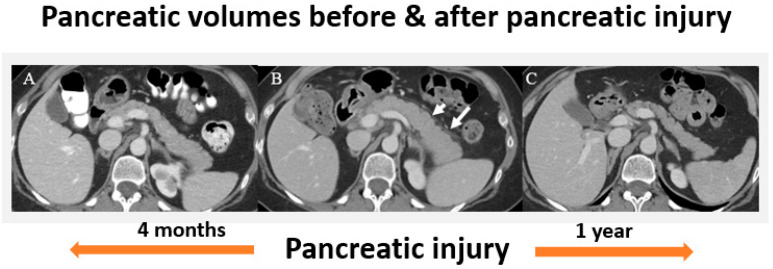
Changes in AIP-3 Over Time on Contrast-Enhanced Computed Tomography (CT). (**A**) Imaging 4 months before ICI-induced pancreatic injury shows a normally appearing pancreas. (**B**) At the time of pancreatic injury, there is diffuse enlargement of the pancreas with peripancreatic stranding/edema (arrows). (**C**) One year after pancreatic injury there is marked pancreatic volume loss without calcification or fatty replacement [15].

The characteristic radiologic patterns and imaging features of AIP-3 remain largely unexplored to date. AIP-3 can appear as the other AIP subtypes or as largely normal on imaging studies. In a retrospective study involving 25 patients receiving ICIs who exhibited evidence of pancreatitis, 56% demonstrated diffuse pancreatic enlargement, while 44% had focal pancreatic enlargement. Heterogeneous enhancement was noted in 84% of cases, and 48% displayed diffuse fat stranding. Additionally, 16% of patients presented with autoimmune pancreatitis featuring focal mass-like pancreatic enlargement. Notably, all cases were classified as mild pancreatitis, with no instances of necrotizing pancreatitis reported [53]. Moreover, the largest study of AIP-3 to date showed that approximately two-thirds of patients had a normal appearing pancreas at the time of diagnosis regardless of their presenting symptoms. The other one-third of patients had imaging features of interstitial pancreatitis, including organ enlargement, loss of the feathery appearance of the pancreas, or peripancreatic stranding/edema [25]. Imaging findings of AIP-3 with the expected changes over time can be seen in Figure 2. In this case, an individual with an initially normal appearing pancreas underwent ICI therapy and developed organ swelling and peripancreatic stranding/edema at the time of pancreatic injury. Repeat imaging one year after the initial injury shows significant pancreatic volume loss consistent with the atrophy that can be expected from chronic pancreatic injury [15]. The enhancing pancreatic rim seen in other forms of AIP is typically not seen in AIP-3 (Table 1). 

**Figure 3:**
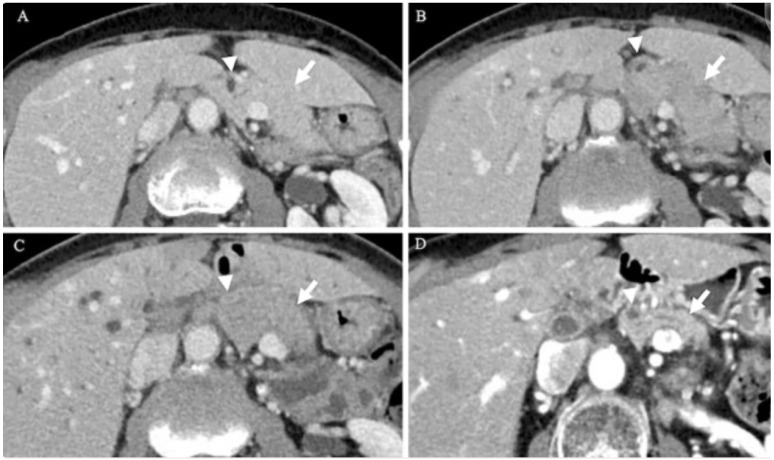
CT scan findings of the pancreas in AIP-3 demonstrated the following changes: (**A**) Prior to ICI treatment, both the pancreas (arrow) and intrapancreatic bile duct (arrowhead) appeared normal. (**B**): Following ICI administration, the pancreas showed diffuse enlargement (arrow), and the intrapancreatic bile duct wall appeared thickened (arrowhead). (**C**) Diffuse enlargement of the pancreas with a capsule-like rim (arrow) and narrowing of the intrapancreatic bile duct (arrowhead) were found. (**D**): The enlargement of the pancreas (arrow) and narrowing of the intrapancreatic bile duct (arrowhead) improved after the administration of steroids [54].

**Figure 4:**
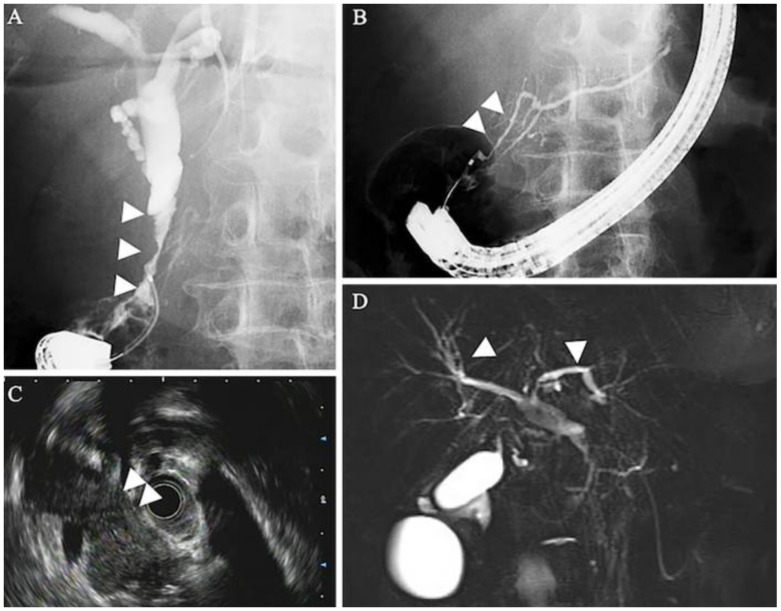
Imaging findings from endoscopic retrograde cholangiopancreatography (ERCP), endoscopic ultrasound (EUS), and magnetic resonance cholangiopancreatography (MRCP) in a patient with AIP-3. (**A**) Endoscopic retrograde cholangiography demonstrated stenosis of the intrapancreatic bile duct (arrowheads). (**B**) Endoscopic retrograde pancreatography revealed narrowing of the main pancreatic duct at the head of the pancreas (arrowheads). (**C**) EUS identified a diffuse hypoechoic enlarged pancreas (arrowheads). (**D**) MRCP showed no evidence of intrahepatic bile duct wall thickening or stenosis [54].

In a separate case of AIP-3, contrast-enhanced CT revealed marked narrowing of the intrapancreatic bile duct, along with wall thickening (Figure 3B,C), dilation of the intrahepatic bile ducts, and diffused pancreatic enlargement (Figure 3C). Endoscopic retrograde cholangiopancreatography (ERCP) confirmed stenosis of the intrapancreatic portion of the common bile duct (Figure 4A) and slight narrowing of the main pancreatic duct at the head of the pancreas (Figure 4B). Endoscopic ultrasound (EUS) revealed a uniformly enlarged and hypoechoic pancreas (Figure 4C). Magnetic resonance cholangiopancreatography (MRCP), however, did not show any signs of thickening or narrowing in the intrahepatic bile ducts (Figure 4D). Notably, the pancreas appeared morphologically normal prior to ICI therapy (Figure 4A), and the patient remained asymptomatic at the time of imaging [54].

### 5.2. Serologic Findings

Elevated serum IgG4 level is considered to be a hallmark of AIP-1. Values above 135–140 mg/dL have been established as the cut-off point for the diagnosis, with variation in sensitivity and specificity according to the population under study [55]. In contrast to AIP-1, there are no specific serologic markers for AIP-2 and AIP-3, and IgG4 levels are usually within normal range in these diseases [9]. However, elevated IgG4 levels have been occasionally described in AIP-3. Additionally, since AIP-2 is associated with IBD in some cases, anti-neutrophil cytoplasmic antibodies (p-ANCA and c-ANCA) may be detected in this subtype [25]. 

### 5.3. Histologic Findings

Histological examination in AIP-1 often reveals dense infiltration of lymphocytes, plasma cells, and occasional eosinophils in the pancreatic parenchyma. The key feature of AIP-1 is the presence of IgG4-positive plasma cells. Diffuse IgG4+ plasma cell infiltration with >10 per high-power field (HPF) in biopsy specimens and >50 per HPF in surgical specimens is strongly suggestive of AIP-1. Additionally, there may be storiform fibrosis (spindle-shaped cells and inflammatory cells on a background of delicate collagen) and obliterative phlebitis (inflammation and occlusion of small veins), which are typical findings of IgG4-related disease [13]. AIP-2 is characterized by an inflammatory infiltrate in the pancreas, predominantly consisting of neutrophils, along with lymphocytes and plasma cells. This inflammation is mainly localized around the pancreatic duct area, where it forms structures called granulocytic epithelial lesions (GELs) [56]. The number of IgG4-positive plasma cells in AIP-2 is generally not markedly elevated, though small clusters of these cells may occasionally be observed [57] (Table 1). 

Currently, there is no universally recognized histologic pattern for AIP-3. However, some general histologic features have been observed in patients with ICI-induced pancreatitis, which distinguish it from the other subtypes. AIP-3 lacks IgG4-positive plasma cell infiltration, storiform fibrosis, and GELs. Instead, there is CD8+ T-cell predominant inflammation with evidence of direct acinar injury. Some cases may exhibit mixed inflammatory infiltrates, including macrophages and CD4+ T cells [58]. While these features provide some guidance, the variability in the clinical presentation and histological findings of AIP-3 underscores the need for more research to establish clearer diagnostic criteria. The current histologic patterns are generally used in conjunction with clinical, imaging, and serologic findings for a comprehensive diagnosis.

## 6. Treatment and Management

Established guidelines for treatment of AIP have indicated that therapy is recommended for symptomatic patients presenting with abdominal pain, back pain, fever, obstructive jaundice, or other organ involvement. For asymptomatic patients, treatment may be indicated if they exhibit a persistent pancreatic mass on imaging or persistent liver test abnormalities in the presence of IgG4-related sclerosing cholangitis [58]. The United European Gastroenterology (UEG) guidelines offer similar recommendations, emphasizing treatment for symptomatic patients and for subclinical conditions that may lead to severe or irreversible organ failure [59]. Recent data suggests that approximately 10–25% of patients with IgG4-RD experience spontaneous symptom resolution without the need for medical treatment [58]. A study by Hart et al., has reported that up to 55% of cases may resolve without intervention [60].

Corticosteroids are the first-line therapy for both AIP-1 and AIP-2. They help inhibit dendritic cell maturation and the downstream signaling of toll-like receptors, stopping the initiation of the innate immune response and blocking several pro-inflammatory cytokines involved in AIP’s pathogenesis. They are also key modulators of the adaptive immune system through their inhibition of lymphocyte activation and promotion of lymphocyte apoptosis [29] (Table 2).

Prednisone is the most commonly used corticosteroid for the treatment of autoimmune pancreatitis (AIP). According to guidelines from the United European Gastroenterology (UEG) and the Swedish Society of Gastroenterology, the recommended initial dose of prednisone is 0.6–0.8 mg/kg per day (typically 30–40 mg daily), which is maintained for one month with clinical assessment after 2–4 weeks of therapy. Following this induction phase, the dose should be gradually tapered by 5 mg per week until a maintenance dose of 2.5–5 mg daily is achieved over a period of 2–3 months. Current literature suggests that maintenance therapy is often continued for 6 months to 3 years in patients at high risk of relapse [54]. Close clinical monitoring is essential to guide tapering and determine the appropriate duration of maintenance treatment. For refractory cases, a mini-pulse steroid regimen involving two courses of methylprednisolone (500 mg/day for three days, with a four-day interval) may be considered [58]. 

Most patients respond well to steroid therapy. Research has shown that up to 90% of patients reach clinical remission, characterized by symptom resolution, normalization of IgG4 levels, and imaging improvement [61,62]. However, it is important to note that relapse can be present in up to 33% of cases [29]. Patients with AIP-1, elevated IgG4 levels, jaundice, and systemic involvement are at higher risk for AIP recurrence [63].

For patients who fail corticosteroid therapy or have contraindications for long-term steroid use, Rituximab has been shown to be an effective single agent in inducing remission and should be considered as the second-line alternative for AIP-1 [64]. According to the 2017 International Consensus Guidelines, immunosuppressive agents, such as azathioprine and mycophenolate mofetil, are useful in inducing remission for select patients with high relapse rates or resistance to corticosteroid therapy. However, they are not effective when used as monotherapy and often require overlap with steroids for 6–8 weeks [28,65]. While these agents offer effective second-line therapy, their use requires careful consideration due to potential adverse effects.

**Table 1:** Key Characteristics and Comparison of Autoimmune Pancreatitis and its Subtypes [9,10,13,14,16,29,37,44,55,56,66,67].

	AIP-1	AIP-2	AIP-3
Demographic	Older males (>60 years)	Younger patients (~50 years)	Patients receiving ICI therapy (age variable)
Clinical Presentation	Painless obstructive jaundice, mild abdominal discomfort, weight loss	Recurrent acute pancreatitis, abdominal pain, jaundice	Asymptomatic or with pancreatitis-like symptoms (abdominal pain, nausea, vomiting) or incidental changes on imaging
Histology	IgG4+ plasma cell infiltration	Granulocytic epithelial lesions (GEL)	CD8 predominant inflammation, often mixed inflammatory infiltrates
Serum IgG4 Levels	Elevated (>135 mg/dL)	Normal	Variable, usually normal
Systemic Involvement	Common (salivary glands, kidneys, lungs, bile ducts, etc.)	Rare	Can be associated with other ICI-related autoimmune conditions
Key Imaging Findings	Diffuse “sausage-shaped” pancreas, capsule-like rim enhancement	Focal/diffuse involvement, strictures without upstream dilation	Normal appearing pancreas, rarely features of interstitial pancreatitis
Response to Steroids	Good response	Good response	Limited data
Risk of Relapse After Treatment	High, requires maintenance therapy	Low	Limited data

**Table 2:** Diagnosis and Management of Autoimmune Pancreatitis [3,9,10,25,29,51,53,55,61,62,64,66].

	AIP-1	AIP-2	AIP-3
Diagnosis and Evaluation	- Elevated serum IgG4 levels (not diagnostic but suggestive) - Imaging showing pancreatic enlargement, peripancreatic rim, ductal strictures - Histopathology (fibrosis, plasma cell infiltration, storiform pattern)	- Imaging showing focal or segmental pancreatic involvement - Serum biomarkers (e.g., normal IgG4 levels, elevated serum amylase/lipase) - presence of granulocytic epithelial lesions (GELs) on histology	- History of ICI therapy - Elevated amylase/lipase levels - Imaging (CT/MRI) to evaluate pancreas
First-Line Treatment	- Prednisone (0.6 mg/kg/day, tapered over 3–6 months) - If high IgG4 levels, initiate therapy promptly	- Prednisone (tapered over 4–6 weeks) - Biologics may be optimal therapy for underlying IBD (anti-TNF, vedolizumab) - Colchicine emerging as possible treatment option	- Discontinuation of ICI therapy
Second-Line Treatment	- Immunosuppressants (azathioprine, mycophenolate mofetil, methotrexate) if relapse or steroid-sparing needed - Rituximab may be used in refractory cases	- Can consider thiopurines or biologics (anti-TNF, vedolizumab) if relapse	- Steroid therapy if needed for refractory cases, but generally not effective
Long-Term Management and Prognosis	- High relapse rate (30–60%); may require long-term low-dose steroids - Immunosuppressants for steroid-sparing management	- Lower relapse rate compared to AIP-1	- Close monitoring for possible biochemical recurrence after ICI reintroduction - Multidisciplinary care with oncologists and rheumatologists
Other Considerations	- Frequent monitoring of IgG4 levels, organ involvement (e.g., lungs, kidneys)	- Associated with IBD, typically UC - Consideration of biologic therapy adjustments if with co-existing IBD	- Close coordination with oncologists for ICI reintroduction decisions - Potential for organ insufficiency requiring long-term monitoring

While steroids remain the first-line therapy for AIP-2, recent studies suggest that colchicine may also be effective as it inhibits neutrophil recruitment and reduces the formation of the pathognomonic GELs [66]. Additionally, biologic agents, such as anti-TNF-alpha and Ustekinumab, have been shown to manage steroid-refractory AIP-2, particularly in patients with coexisting IBD [9] (Table 2).

The most important aspect of AIP-3 treatment is early recognition of the condition and discontinuation of the offending agent. Unlike classical AIP, the role of corticosteroid therapy in AIP-3 remains controversial due to current studies not showing significant improvement in duration of symptoms or decrease in risk of hospitalization [10]. One such study observed that treatment with corticosteroids did not have any effect on severity of symptoms, mitigating pancreatic volume loss, or in inducing and maintaining remission [15]. Additionally, corticosteroid use in immunocompromised patients also increases the risk of infection and this should be taken into consideration prior to starting corticosteroids in any patient receiving immunosuppression. Various supportive measures and symptom management may also be indicated in AIP-3, including pancreatic enzyme supplementation. Approximately 44% of patients with AIP-3 develop a degree of pancreatic parenchymal atrophy and a significant portion of these individuals go on to develop pancreatic insufficiency. In this population, pancreatic enzyme replacement should be considered to alleviate symptoms, such as steatorrhea. Little is known about consequent endocrine insufficiency in this autoimmune process (Table 2).

Ultimately, further studies are needed to create universal guidelines for treatment of AIP-3. However, while data supporting the benefit of corticosteroid therapy is minimal, it would be reasonable to consider corticosteroid initiation in clinical practice for individuals with severe grade AIP-3 or in those with no improvement from supportive therapies [10,56].

## 7. Conclusions

In conclusion, AIP is a rare, complex condition characterized by inflammation of the pancreas secondary to an abnormal immune response. It is classified by three subtypes: Type 1 (AIP-1), Type 2 (AIP-2), and Type 3 (AIP-3). Each type of AIP is characterized by different pathophysiologic, clinical, radiographic, and histologic features that aid in proper diagnosis. AIP-1, or IgG4-related pancreatitis, is recognized by the presence of lymphoplasmacytic infiltration of the pancreas with the potential for systemic involvement, while the inflammation in AIP-2 is not systemic and is localized to the pancreas. AIP-3 is unique among the autoimmune pancreatitides in that it is a rare side effect of immune checkpoint inhibitor therapy. Given the varying methods of treatment for the different types of AIP, proper awareness and diagnosis of the condition is crucial. Furthermore, as ICI use becomes more prevalent, research regarding AIP-3 will be essential to understand its pathogenesis, create guidelines for diagnosis, and standardize the approach to treatment.

## References

[1] Blaho M., Dítě P., Kunovský L., Kianička B. (2020). Autoimmune pancreatitis—An ongoing challenge. Adv. Med. Sci..

[2] Vujasinovic M., Löhr J.M., Domínguez-Muñoz J.E. (2021). Autoimmune pancreatitis: Definition, clinical presentation, and classification. Clinical Pancreatology for Practising Gastroenterologists and Surgeons.

[3] Takahashi M., Fujinaga Y., Notohara K., Koyama T., Inoue D., Irie H., Gabata T., Kadoya M., Kawa S., Okazaki K. (2020). Diagnostic imaging guide for autoimmune pancreatitis. Jpn. J. Radiol..

[4] Nikolic S., Maisonneuve P., Dahlman I., Löhr J.-M., Vujasinovic M. (2022). Exocrine and endocrine insufficiency in autoimmune pancreatitis: A matter of treatment or time?. J. Clin. Med..

[5] Qureshi A., Ghobrial Y., De Castro J., Siami-Namini K., Newman K.A. (2021). Autoimmune pancreatitis—What we know and what do we have to know?. Autoimmun. Rev..

[6] Sandrasegaran K., Menias C.O. (2018). Imaging in autoimmune pancreatitis and immunoglobulin g4–related disease of the abdomen. Gastroenterol. Clin. N. Am..

[7] Khandelwal A., Inoue D., Takahashi N. (2020). Autoimmune pancreatitis: An update. Abdom. Radiol..

[8] Nan N., Wang D. (2023). Type 2 autoimmune pancreatitis associated with ulcerative colitis. Front. Immunol..

[9] De Pretis N., Frulloni L. (2020). Autoimmune pancreatitis type 2. Curr. Opin. Gastroenterol..

[10] Sayed Ahmed A., Abreo M., Thomas A., Chari S.T. (2022). Type 3 autoimmune pancreatitis (immune checkpoint inhibitor-induced pancreatitis). Curr. Opin. Gastroenterol..

[11] Tan S., Day D., Nicholls S.J., Segelov E. (2022). Immune checkpoint inhibitor therapy in oncology. JACC CardioOncol..

[12] Ramos-Casals M., Brahmer J.R., Callahan M.K., Flores-Chávez A., Keegan N., Khamashta M.A., Lambotte O., Mariette X., Prat A., Suárez-Almazor M.E. (2020). Immune-related adverse events of checkpoint inhibitors. Nat. Rev. Dis. Primers.

[13] Gallo C., Dispinzieri G., Zucchini N., Invernizzi P., Massironi S. (2024). Autoimmune pancreatitis: Cornerstones and future perspectives. World J. Gastroenterol..

[14] Liu Y., Zhang H., Zhou L., Li W., Yang L., Li W., Li K., Liu X. (2021). Immunotherapy-associated pancreatic adverse events: Current understanding of their mechanism, diagnosis, and management. Front. Oncol..

[15] Thomas A.S., Abreo M., Ahmed A.S., Rao Manikonda S.P., Eyada M., Issac A., Abraham F., Jacob J.S., Wang Y., Yedururi S. (2024). Immune checkpoint inhibitor-induced pancreatic injury: Clinical and radiological profile and response to steroids. Gastro Hep Adv..

[16] Basyal B., Kc P. (2025). Autoimmune pancreatitis. StatPearls.

[17] Schneider A., Michaely H., Weiss C., Hirth M., Rückert F., Wilhelm T.J., Schönberg S., Marx A., Singer M.V., Löhr J.M. (2017). Prevalence and incidence of autoimmune pancreatitis in the population living in the southwest of Germany. Digestion.

[18] Masamune A., Kikuta K., Hamada S., Tsuji I., Takeyama Y., Shimosegawa T., Okazaki K. (2020). Nationwide epidemiological survey of autoimmune pancreatitis in Japan in 2016. J. Gastroenterol..

[19] Kanno A., Masamune A., Okazaki K., Kamisawa T., Kawa S., Nishimori I., Tsuji I., Shimosegawa T. (2015). Nationwide epidemiological survey of autoimmune pancreatitis in Japan in 2011. Pancreas.

[20] Li Y., Song H., Meng X., Li R., Leung P.S.C., Gershwin M.E., Zhang S., Sun S., Song J. (2023). Autoimmune pancreatitis type 2 (Idiopathic duct-centric pancreatitis): A comprehensive review. J. Autoimmun..

[21] George J., Bajaj D., Sankaramangalam K., Yoo J.W., Joshi N.S., Gettinger S., Price C., Farrell J.J. (2019). Incidence of pancreatitis with the use of immune checkpoint inhibitors (ICI) in advanced cancers: A systematic review and meta-analysis. Pancreatology.

[22] Abu-Sbeih H., Tang T., Lu Y., Thirumurthi S., Altan M., Jazaeri A.A., Dadu R., Coronel E., Wang Y. (2019). Clinical characteristics and outcomes of immune checkpoint inhibitor-induced pancreatic injury. J. Immunother. Cancer.

[23] Wang Y. (2022). Managing Immunotherapy Related Organ Toxicities: A Practical Guide.

[24] Shirwaikar Thomas A., Chari S.T. (2023). Immune checkpoint inhibitor-induced (Type 3) autoimmune pancreatitis. Curr. Gastroenterol. Rep..

[25] Thomas A.S., Abreo M., Sayed S.A., Sireesha Yedururi Y.W., Chari S.T. (2023). Autoimmune pancreatitis secondary to immune checkpoint inhibitor therapy (Type 3 AIP): Insights into a new disease from serial pancreatic imaging. Gastroenterology.

[26] Wang N., Zhu P., Xiang Y., Tao L., Huang T., Feng Z. (2024). IgG4-related autoimmune pancreatitis and sclerosing cholangitis: A case report and literature review. Medicine.

[27] Kamisawa T., Zen Y., Pillai S., Stone J.H. (2015). IgG4-related disease. Lancet.

[28] Kokubo K., Onodera A., Kiuchi M., Tsuji K., Hirahara K., Nakayama T. (2022). Conventional and pathogenic Th2 cells in inflammation, tissue repair, and fibrosis. Front. Immunol..

[29] Nista E.C., De Lucia S.S., Manilla V., Schepis T., Pellegrino A., Ojetti V., Pignataro G., Zileri Dal Verme L., Franceschi F., Gasbarrini A. (2022). Autoimmune pancreatitis: From pathogenesis to treatment. Int. J. Mol. Sci..

[30] Minaga K., Watanabe T., Hara A., Yoshikawa T., Kamata K., Kudo M. (2021). Plasmacytoid dendritic cells as a new therapeutic target for autoimmune pancreatitis and IgG4-related disease. Front. Immunol..

[31] Psarras A., Emery P., Vital E.M. (2017). Type I interferon–mediated autoimmune diseases: Pathogenesis, diagnosis and targeted therapy. Rheumatology.

[32] Ku Y., Hong S.-M., Fujikura K., Kim S.J., Akita M., Abe-Suzuki S., Shiomi H., Masuda A., Itoh T., Azuma T. (2017). IL-8 expression in granulocytic epithelial lesions of idiopathic duct-centric pancreatitis (type 2 autoimmune pancreatitis). Am. J. Surg. Pathol..

[33] Loos M., Lauffer F., Schlitter A.M., Kleeff J., Friess H., Klöppel G., Esposito I. (2015). Potential role of Th17 cells in the pathogenesis of type 2 autoimmune pancreatitis. Virchows Arch..

[34] Osaki M., Sakaguchi S. (2025). Soluble CTLA-4 regulates immune homeostasis and promotes resolution of inflammation by suppressing type 1 but allowing type 2 immunity. Immunity.

[35] Zhang M., Ni J., Xu W.-D., Wen P.-F., Qiu L.-J., Wang X.-S., Pan H.-F., Ye D.-Q. (2014). Association of CTLA-4 variants with susceptibility to inflammatory bowel disease: A meta-analysis. Hum. Immunol..

[36] Klocke K., Sakaguchi S., Holmdahl R., Wing K. (2016). Induction of autoimmune disease by deletion of CTLA-4 in mice in adulthood. Proc. Natl. Acad. Sci. USA.

[37] Lanzillotta M., Vujasinovic M., Löhr J., Della Torre E. (2024). Update on autoimmune pancreatitis and igg4-related disease. United Eur. Gastroenterol. J..

[38] Xu B., Cai Z.-P., Liu W. (2023). IgG4-related cholangiopathy, pancreatopathy and lymphadenopathy. Am. J. Med. Sci..

[39] Wu S., Wang H. (2023). IgG4-related digestive diseases: Diagnosis and treatment. Front. Immunol..

[40] Li M.-Z., Guo T., Feng Y.-L., Zhang S.-Y., Bai X.-Y., Wu X., Xu K., Yang A.-M. (2024). Diabetes mellitus in patients with type 1 autoimmune pancreatitis at diagnosis and after corticosteroid therapy. Hepatobiliary Pancreat. Dis. Int..

[41] Noguchi K., Nakai Y., Mizuno S., Isayama H., Hirano K., Kanai S., Nakamura T., Uchino R., Takahara N., Kogure H. (2020). Insulin secretion improvement during steroid therapy for autoimmune pancreatitis according to the onset of diabetes mellitus. J. Gastroenterol..

[42] Matsushiro M., Haraguchi T., Yamazaki Y., Hamamoto Y., Seino Y. (2025). Effects of steroid therapy on pancreatic endocrine function in IgG4-related aip: Evaluation by arginine stimulation test. JCEM Case Rep..

[43] Nishimori I., Tamakoshi A., Kawa S., Tanaka S., Takeuchi K., Kamisawa T., Saisho H., Hirano K., Okamura K., Yanagawa N. (2006). Research Committee on Intractable Pancreatic Diseases, the Ministry of Health and Welfare of Japan. Influence of steroid therapy on the course of diabetes mellitus in patients with autoimmune pancreatitis: Findings from a nationwide survey in Japan. Pancreas.

[44] Hart P.A., Levy M.J., Smyrk T.C., Takahashi N., Abu Dayyeh B.K., Clain J.E., Gleeson F.C., Pearson R.K., Petersen B.T., Topazian M.D. (2016). Clinical profiles and outcomes in idiopathic duct-centric chronic pancreatitis (type 2 autoimmune pancreatitis): The Mayo Clinic experience. Gut.

[45] Massironi S., Fanetti I., Viganò C., Pirola L., Fichera M., Cristoferi L., Capurso G., Invernizzi P., Danese S. (2022). Systematic review—Pancreatic involvement in inflammatory bowel disease. Aliment. Pharmacol. Ther..

[46] Lorenzo D., Maire F., Stefanescu C., Gornet J.-M., Seksik P., Serrero M., Bournet B., Marteau P., Amiot A., Laharie D. (2018). Features of autoimmune pancreatitis associated with inflammatory bowel diseases. Clin. Gastroenterol. Hepatol..

[47] Banks P.A., Bollen T.L., Dervenis C., Gooszen H.G., Johnson C.D., Sarr M.G., Tsiotos G.G., Vege S.S., Acute Pancreatitis Classification Working Group (2013). Classification of acute pancreatitis—2012: Revision of the Atlanta classification and definitions by international consensus. Gut.

[48] Shimosegawa T., Beger H.G., Büchler M.W., Hruban R.H., Mayerle J., Neoptolemos J.P., Shimosegawa T., Warshaw A.L., Whitcomb D.C., Zhao Y., Groß C. (2023). Clinical diagnostic criteria for autoimmune pancreatitis. The Pancreas.

[49] Maruyama M., Watanabe T., Kanai K., Oguchi T., Muraki T., Hamano H., Arakura N., Kawa S. (2013). International consensus diagnostic criteria for autoimmune pancreatitis and its japanese amendment have improved diagnostic ability over existing criteria. Gastroenterol. Res. Pract..

[50] Rehnitz C., Klauss M., Singer R., Ehehalt R., Werner J., Büchler M.W., Kauczor H.-U., Grenacher L. (2011). Morphologic patterns of autoimmune pancreatitis in CT and MRI. Pancreatology.

[51] Lee S., Kim J.H., Kim S.Y., Byun J.H., Kim H.J., Kim M.-H., Lee M.-G., Lee S.S. (2018). Comparison of diagnostic performance between CT and MRI in differentiating non-diffuse-type autoimmune pancreatitis from pancreatic ductal adenocarcinoma. Eur. Radiol..

[52] O’Reilly D.A., Malde D.J., Duncan T., Rao M., Filobbos R. (2014). Review of the diagnosis, classification and management of autoimmune pancreatitis. World J. Gastrointest. Pathophysiol..

[53] Das J.P., Postow M.A., Friedman C.F., Do R.K., Halpenny D.F. (2020). Imaging findings of immune checkpoint inhibitor associated pancreatitis. Eur. J. Radiol..

[54] Tanabe K., Yokoyama K., Kanno A., Ikeda E., Ando K., Nagai H., Koyanagi T., Sakaguchi M., Nakaya T., Tamada K. (2024). Immune checkpoint inhibitor-induced pancreatitis with pancreatic enlargement mimicking autoimmune pancreatitis: A case report and review of the literature. Intern. Med..

[55] Yokode M., Shiokawa M., Kodama Y. (2021). Review of diagnostic biomarkers in autoimmune pancreatitis: Where are we now?. Diagnostics.

[56] Chen L., Orr C.E., Wang T. (2020). Prevalence of histological features resembling autoimmune pancreatitis in neoplastic pancreas resections. Histopathology.

[57] Deshpande V., Gupta R., Sainani N., Sahani D.V., Virk R., Ferrone C., Khosroshahi A., Stone J.H., Lauwers G.Y. (2011). Subclassification of autoimmune pancreatitis: A histologic classification with clinical significance. Am. J. Surg. Pathol..

[58] Okazaki K., Chari S.T., Frulloni L., Lerch M.M., Kamisawa T., Kawa S., Kim M.-H., Lévy P., Masamune A., Webster G. (2017). International consensus for the treatment of autoimmune pancreatitis. Pancreatology.

[59] Löhr J.-M., Beuers U., Vujasinovic M., Alvaro D., Frøkjær J.B., Buttgereit F., Capurso G., Culver E.L., de-Madaria E., Della-Torre E. (2020). European Guideline on IgG4-related digestive disease—UEG and SGF evidence-based recommendations. United Eur. Gastroenterol. J..

[60] Hart P.A., Kamisawa T., Brugge W.R., Chung J.B., Culver E.L., Czakó L., Frulloni L., Go V.L.W., Gress T.M., Kim M.-H. (2013). Long-term outcomes of autoimmune pancreatitis: A multicentre, international analysis. Gut.

[61] Matsubayashi H., Yoneyama M., Nanri K., Sugimoto S., Shinjo K., Kakushima N., Tanaka M., Ito S., Takao M., Ono H. (2013). Determination of steroid response by abdominal ultrasound in cases with autoimmune pancreatitis. Dig. Liver Dis..

[62] Matsubayashi H., Ishiwatari H., Imai K., Kishida Y., Ito S., Hotta K., Yabuuchi Y., Yoshida M., Kakushima N., Takizawa K. (2019). Steroid therapy and steroid response in autoimmune pancreatitis. Int. J. Mol. Sci..

[63] Tacelli M., Celsa C., Magro B., Barresi L., Guastella S., Capurso G., Frulloni L., Cabibbo G., Cammà C. (2019). Risk factors for rate of relapse and effects of steroid maintenance therapy in patients with autoimmune pancreatitis: Systematic review and meta-analysis. Clin. Gastroenterol. Hepatol..

[64] Akiyama M., Takeuchi T. (2018). IgG4-related disease: Beyond glucocorticoids. Drugs Aging.

[65] Brito-Zerón P., Kostov B., Bosch X., Acar-Denizli N., Ramos-Casals M., Stone J.H. (2016). Therapeutic approach to IgG4-related disease: A systematic review. Medicine.

[66] Chiabrando F., Lanzillotta M., Palumbo D., Pedica F., Caruso M., Capurso G., Della-Torre E. (2021). Treating type 2 autoimmune pancreatitis with colchicine: A case series. Ann. Intern. Med..

[67] Satish D., Lin I.-H., Flory J., Gerdes H., Postow M.A., Faleck D.M. (2023). Exocrine pancreatic insufficiency induced by immune checkpoint inhibitors. Oncologist.

